# Therapeutic Effects of *Psoralea corylifolia* and *Morus alba* Aqueous Extracts on *Tetrahymena pyriformis*-Infected Guppies (*Poecilia reticulata*) and Underlying Transcriptomic Mechanisms: Implications for Ciliate Parasite Control

**DOI:** 10.3390/ani16060979

**Published:** 2026-03-20

**Authors:** Sitong Li, Pengfei Zhang, Yunhan Wang, Yuxuan Wang, Huan Li, Xuming Pan

**Affiliations:** 1 Key Laboratory of Biodiversity of Aquatic Organisms, Harbin Normal University, Harbin 150025, China; 2 College of Teacher Education, Harbin Normal University, Harbin 150025, China

**Keywords:** phytotherapy, ciliated protozoa, antiparasitic activity, gene expression, aquatic disease control

## Abstract

*Tetrahymena pyriformis* is biologically similar to *Ichthyophthirius multifiliis*, the parasite that causes “white spot disease” in fish. Because it has immune evasion genes and grows quickly, *T. pyriformis* serves as an ideal model for developing treatments against *I. multifiliis* and related parasites. This study tested water extracts from 10 traditional Chinese herbs against *T. pyriformis* and identified 5 with strong antiparasitic effects: *Morus alba*, *Psoralea corylifolia*, *Sophora flavescens*, *Polygonum cuspidatum*, and Pomegranate Peel. Combination tests showed that certain pairs, especially *P. corylifolia* with *M. alba*, worked together synergistically. When infected guppies were treated with this herbal combination at a concentration of 1.39 g/L (1:144 dilution), their 10-day survival rate reached 66.7%. Gill tissue analysis identified 577 genes with changed activity after treatment—228 increased and 349 decreased. These genes were linked to immune responses, metabolism, and cell processes. The key differentially expressed genes include those involved in the IL-17 signaling pathway, amino sugar metabolism, and the cytosolic DNA-sensing pathway. These results show that the herbal combination works by both directly killing parasites and boosting the fish’s immune system. This study provides a scientific basis for using natural herbal treatments as an eco-friendly way to control parasitic diseases in aquaculture.

## Simple Summary

Fish farming faces serious losses from parasites like the one causing “white spot disease.” This study tested whether natural Chinese herbs could help protect fish from these tiny invaders. Researchers mixed extracts from ten different herbs and found that a combination of two—*Psoralea corylifolia* and *Morus alba*—worked best together to kill the parasites. When sick guppies were given a mild bath in this herbal mixture, most survived, while nearly all untreated fish died. The treatment not only killed the parasites directly but also helped the fish defend themselves. By studying the fish’s gill tissue, scientists discovered that the herbs boosted the fish’s own immune system and changed how their cells used energy to fight infection. This meant the fish could better resist the parasites and recover from damage. Using plant-based treatments like this offers a safer, natural alternative to chemical drugs, which can harm the environment and lead to drug-resistant parasites. This approach could help fish farmers keep their stocks healthy in a more sustainable way.

## 1. Introduction

*Tetrahymena pyriformis* is a species of ciliated protozoan commonly found in freshwater environments, taxonomically belonging to the class Oligohymenophorea and subclass Hymenostomatia. *T. pyriformis* is morphologically related to *Ichthyophthirius multifiliis* [1]. As a facultative parasite, it is frequently present in closed aquatic environments or parasitizes injured aquatic fish [2,3,4]. As an opportunistic pathogen, *T. pyriformis* often invades the epithelial tissues of fish hosts when their body surface is injured or secondary to other pathogenic infections [4,5]. In severe cases, *T*. *pyriformis* can attach in large numbers to the surface of gill filaments, impeding gas exchange and even causing host death [3]. Unlike *I. multifiliis* and *Cryptocaryon irritans*, which have a two-stage life cycle consisting of trophozoite and cyst stages [6], *T. pyriformis* has a single morphological life cycle and is capable of independent and rapid proliferation outside the host [7,8]. Although extensive research has been conducted on its taxonomy, toxicology, physiology, and genetics [9,10,11], the immune response mechanism of fish to Chinese herbal medicine treatment after infection with this protozoan remains unclear.

The guppy (*Poecilia reticulata*), native to South America, is one of the most popular ornamental fish worldwide due to its vibrant coloration and adaptability [12]. As a live-bearing species with a short generation time, guppies serve as excellent biological models for parasitological research, particularly in studies of host–parasite coevolution and disease susceptibility [13,14]. However, intensive aquaculture has exacerbated disease outbreaks, with ciliate parasites such as *I. multifiliis* and *T. pyriformis* causing massive mortality by invading gills and disrupting respiration [15,16]. The limited therapeutic options available underscore the urgent need for effective, environmentally sustainable disease control strategies.

Current conventional control methods for ciliate diseases still rely mainly on chemical drugs, such as copper sulfate, potassium permanganate, formalin, and various disinfectants [17,18,19]. However, long-term or excessive use of these drugs easily induces parasite resistance and causes problems such as drug residues, ecological environment pollution, and toxicity to fish [20,21,22]. Therefore, exploring safe and sustainable alternative prevention and control methods is particularly important. Chinese herbal medicines have gradually become a research hotspot in the field of aquatic parasite control due to their good environmental compatibility, biodegradability, and diverse pharmacological activities [23,24]. It has been reported that certain plant-derived components, such as Isobavachalcone from *Psoralea corylifolia* [25], cynatratoside-C from *Cynanchum atratum* [26], magnolol from *Magnolia officinalis* [27], 10-gingerol from *Zingiber officinale* [28], sophoraflavanone G from *Sophora flavescens* [29], epicatechin gallate from *Sargentodoxa cuneata* [30], as well as kuwanon G and kuwanon O from the root bark of *Morus alba* [31], all exhibit anti-ciliate activity. These natural products are regarded as important sources for the development of new antiparasitics due to their multi-target mode of action and low environmental impact. However, existing studies mainly focus on their direct lethal effect on protozoa, and in-depth research on the mechanism by which they enhance anti-infection ability by regulating the host immune system is still lacking [32,33].

Plant-derived antiparasitic agents face a fundamental challenge: the narrow therapeutic index. Many bioactive phytochemicals exhibit potent toxicity to both parasites and host organisms. For instance, *Psoralea corylifolia* contains psoralidin and isopsoralen, compounds with significant antiparasitic activity against *I. multifiliis* but also potential hepatotoxicity, resulting in a narrow safety margin [34]. Recent studies have confirmed that psoralen, isopsoralen, and bakuchiol are the main bioactive components, with hepatotoxicity being the predominant adverse effect in mammalian models [35]. Similarly, other antiparasitic plant metabolites frequently display non-selective cytotoxicity, causing gill epithelial damage at concentrations required for effective parasite elimination [36]. Therefore, identifying synergistic botanical combinations is crucial not only for enhancing efficacy but also for reducing individual component dosages to mitigate host toxicity [37,38]. This “synergistic toxicity reduction” strategy represents a critical paradigm in aquatic phytotherapy [37].

Recent advances in teleost mucosal immunology have provided crucial insights into how fish defend against ciliate infections at epithelial surfaces. The gill serves as the front line of host–parasite interaction [39]. Immunological investigations have elucidated that IL-17 responses play pivotal roles in the gill epithelium during infections [40]. Notably, IL-17 receptors show broad expression in fish mucosal tissues, with the highest expression in gills, skin, and intestine [41]. Upon recognition of pathogen-associated molecular patterns (PAMPs), teleost gill epithelial cells activate the IL-17 signaling pathway, promoting the release of inflammatory cytokines such as IL-6 and TNF-α [40,42]. Additionally, the cytosolic DNA-sensing cGAS-STING pathway has been increasingly implicated in defense against protozoan parasites through the detection of foreign nucleic acids [43]. Furthermore, metabolic reprogramming—particularly mucin glycoprotein synthesis—has emerged as a critical component of mucosal defense, directly influencing parasite adhesion [39]. These molecular mechanisms establish a theoretical framework for understanding how plant extracts may enhance host anti-parasitic capacity through immunomodulation and metabolic support.

Therefore, this study used ciliates with typical facultative parasitic characteristics as biological models to systematically screen plant extracts with anti-parasitic activity and explore their combined effects. By establishing an in vivo infection model, the impact of the optimized combination on the survival status of the host was evaluated, and high-throughput transcriptome technology was used to analyze the immune response and metabolic remodeling process of host tissues under drug intervention. This study aims to clarify the potential pathways by which plant extracts enhance anti-parasitic ability through host-oriented mechanisms, and provide a theoretical basis for the development of aquaculture parasite control strategies based on immune regulation.

## 2. Materials and Methods

### 2.1. Fish and Parasites

A total of 500 healthy guppies (*Poecilia reticulata*) used in the experiment were purchased from an ornamental fish market in Harbin City. The fish had a body length of 4.1 ± 0.2 cm (mean ± standard deviation) and an average body weight of 10 ± 1.2 g. They were fed commercial fish feed pellets (crude protein ≥ 55%, crude fat ≥ 8%, crude fiber ≤ 3%, crude ash ≤ 16%, moisture ≤ 9%, total phosphorus 1.5–3%, lysine ≥ 2.5%) once daily at a feeding rate of 1% of body weight at 5:00 p.m. All experimental fish were reared in circular aquariums with a volume of 1 m^3^ (100 cm × 100 cm × 100 cm) and placed in an air-conditioned, temperature-controlled room. The water temperature was maintained at 25.1 ± 0.6 °C, and the pH value was 7.1 ± 0.4. The dissolved oxygen content was controlled within the range of 5.0–7.0 mg/L (measured value: 6.1 ± 0.58 mg/L), with ammonia nitrogen at 0.16 ± 0.09 mg/L and nitrite nitrogen at 0.18 ± 0.01 mg/L. Fish were acclimated for 14 days prior to experimentation. The entire in vivo antiparasitic efficacy evaluation was conducted over a 10-day observation period post-treatment.

Ciliates were initially isolated from naturally infected guppies in a fish farm in Harbin. Under a stereomicroscope, multiple pear-shaped *T. pyriformis* cells were isolated from the gills and mucus of infected guppies using a sterile micropipette. Monoclonal cultures of *T. pyriformis* were established in 20 mL Petri dishes using sterile RM-9 medium and incubated at 25 °C [5]. All parasite isolation and culture procedures were carried out under aseptic conditions using sterile micropipettes, Petri dishes, and culture media to prevent contamination.

### 2.2. Preparation of Chinese Herbal Medicines and Their Aqueous Extracts

All Chinese herbal medicine raw materials used in this study were dried plant materials (purchased from Bozhou Kangmei Chinese Medicine City, Bozhou, China). Deionized water was added at a ratio of 20% (*W*/*V*, weight of dried Chinese herbal medicine/volume of deionized water), and the mixture was soaked for 45 min to 1 h to allow the medicinal materials to fully absorb water and swell. Subsequently, the mixture was heated to boiling and maintained at a gentle boil for a certain period. After decoction, the filtrate was collected by hot filtration through gauze, and the dregs were decocted twice. All filtrates were combined as the stock solution. The stock solution had a concentration of 200 g/L (based on 20% *W*/*V*, i.e., 20 g dried herb per 100 mL water). The concentrations required for the experiment were obtained by diluting the stock solution with deionized water and are expressed as the final concentration (g/L) of the herbal extract in the test solution. Freshly prepared Chinese herbal extracts were used in each experiment.

### 2.3. Antiparasitic Screening of Aqueous Extracts from 10 Chinese Herbal Medicines

Aqueous extracts of 10 Chinese herbal medicines were prepared using the aforementioned extraction method. In each well of a 24-well cell culture plate, 100 μL of water containing approximately 1 × 10^4^ *T. pyriformis* cells was added, followed by 100 μL of the test solution of Chinese herbal extracts at final concentrations of 100, 50, 25, 12.5, 6.25, 3.125, and 0 g/L (corresponding to 1:2, 1:4, 1:8, 1:16, 1:32, 1:64, and 0 dilutions of the stock solution, respectively). Each concentration was performed in triplicate. Within a 5 h period after treatment, each well was observed regularly under a microscope to determine the mortality rate of *T. pyriformis* by checking for loss of motility and abnormal morphology. Due to the high cell density (~10^4^ cells per well), individual cell tracking was not feasible; therefore, mortality was assessed at the population level as the time required to achieve 100% mortality in each well. Extracts with strong antiparasitic activity were selected for further experiments.

### 2.4. Checkerboard Assay

The extracts of *Morus alba* (white mulberry root bark), *Psoralea corylifolia* (Psoralea fruit), *Sophora flavescens* (lightyellow sophora root), *Polygonum cuspidatum* (Japanese knotweed), and Pomegranate Peel exhibited high efficacy against parasites. Therefore, these five plants were selected to study their pairwise interactions using the checkerboard assay [44]. The Minimum Lethal Concentration (MLC), defined as the concentration that killed 100% of the trophozoites after 5 h of exposure, was determined in the antiparasitic screening experiment. Each plant extract was prepared at concentrations of 2 MLC, 1 MLC, 0.5 MLC, and 0.25 MLC using a two-fold dilution method. Four dilutions of plant extract A were mixed with four dilutions of plant extract B at a ratio of 1:1 (*V*:*V*), resulting in 16 mixtures (100 μL per mixture), each containing a unique combination of plant A and plant B. Then, 100 μL of each mixture was mixed with water containing approximately 1 × 10^4^ *T. pyriformis* cells. The final concentrations of plant A or B used (0.5 MLC, 0.25 MLC, 0.125 MLC, and 0.06 MLC) were four times lower than the initial concentrations (2 MLC, 1 MLC, 0.5 MLC, and 0.25 MLC). The Fractional Inhibitory Concentration (FIC) index was calculated as follows: FIC index = (MIC of A in combination/MIC of A alone) + (MIC of B in combination/MIC of B alone). Drug interactions were defined according to established pharmacological criteria: Synergism, FIC ≤ 0.5; Additive effect, 0.5 < FIC ≤ 1; Indifference, 1 < FIC ≤ 4; Antagonism, FIC > 4. Synergism refers to an effect where the combined action is greater than the sum of the individual effects; additive effect refers to a combined result equal to the sum of the individual component effects; indifference refers to an effect less than the individual component effects; and antagonism refers to a negative interaction where the efficacy of one bioactive compound is reduced by other substances [45].

### 2.5. Preparation of Plant Combinations with Synergistic and Additive Effects

Based on the results of the checkerboard assay, plant combinations with synergistic and additive effects were selected for further study. According to the aforementioned method, 50 g of each plant combination (mass ratio of plant A to plant B = 1:1) was dried and decocted for extraction. Mass ratios were selected for practical production applications, with preliminary observations indicating comparable extraction efficiencies between the two botanical materials. Freshly prepared Chinese herbal extracts were used in each experiment.

### 2.6. Antiparasitic Effects of Plant Combinations

In 24-well plates, the stock solution of each plant combination was serially diluted to obtain final concentrations of 100, 50, 25, 12.5, 6.25, 3.125, and 0 g/L (corresponding to 1:2, 1:4, 1:8, 1:16, 1:32, 1:64, and 0 dilutions) after adding 100 μL of parasite suspension. The actual test concentrations after mixing were 50, 25, 12.5, 6.25, 3.125, 1.56, and 0 g/L. Each concentration was set in triplicate. All *T. pyriformis* cells were continuously monitored for the duration until death within 5 consecutive hours. Loss of motility was considered as death.

### 2.7. In Vivo Antiparasitic Efficacy Evaluation of Aqueous Extracts from Plant Combinations

The antiparasitic experiment results of plant combinations showed that Combination 6 (Com 6: *P. corylifolia* + *M. alba*) exhibited the highest antiparasitic activity against *T. pyriformis* in vitro. Therefore, Combination 6 was used for further in vivo experiments.

The in vivo infection model was established using healthy guppies, which were confirmed free of parasitic infections through microscopic examination [46]. Infection of healthy guppies with *T. pyriformis* was performed using the scratch inoculation method at an infection dose of 50 cells/mL [47]. To accelerate the infection process, infected fish were co-reared with healthy fish in 1 m^3^ glass containers. When the fish reached the moderate infection standard (approximately 20 *T. pyriformis* cells parasitic per fin), 180 infected fish were randomly divided into 18 groups (10 fish per group) and placed in plastic buckets with a volume of 2000 cm^3^, each containing 1 L of aerated tap water. Meanwhile, 30 healthy guppies were randomly divided into 3 groups (10 fish per group) and placed in plastic buckets of the same specification with the same water conditions as the blank control group.

A preliminary experiment was conducted to determine the tolerance of guppies to the water extract of Chinese herbal medicine. The results showed that at a concentration of 1:8, fish exhibited hypoxia symptoms such as floating after 10 min of immersion. At a concentration of 1:16, the same symptoms were observed after 20 min of immersion. At concentrations of 6.25 g/L and 3.125 g/L (corresponding to 1:32 and 1:64 dilutions), fish showed slowed movement after four hours of immersion, but all died the following day. Finally, a concentration of 1.56 g/L (1:128 dilution) was used, and a 5 h immersion treatment was administered to the infected guppies. It was found that this concentration effectively eliminated most parasites, and the health of the experimental fish remained good. However, extending the immersion time or increasing the extract concentration led to a deterioration in the fish’s condition, with more than half of the experimental fish dying within 24 h. Therefore, 180 infected fish were randomly assigned to aqueous immersion therapy with Chinese herbal medicine solutions at final concentrations of 1.56, 1.39, 1.25, 1.14, 1.04, and 0 g/L for 5 h, followed by transfer to normal aerated tap water. All infection experiments were conducted at 25.0 ± 1 °C with three replicates. The 6 fish used for RNA-seq analysis were included in the survival count, and the total number of fish per treatment group was 30.

### 2.8. Gill Tissue Sampling, RNA Extraction, Library Construction, and Sequencing

All experiments were performed in accordance with the Guide for the Care and Use of Laboratory Animals of the National Institutes of Health (NIH Publication No. 85-23, Revised 1985) and approved by the Ethics Committee of Harbin Normal University. The survival number and status of the fish were observed every 6 h. In the untreated group, nearly half of the fish died after 72 h. On the third day, based on the in vivo antiparasitic results, fish from the concentration group with the highest survival rate (Treatment group, T) and untreated infected fish (Control group, C) were selected for sampling. Before dissection, the fish were anesthetized with MS-222, and the gills of all surviving fish were collected and rinsed with cold DEPC water to remove ciliates on the surface of the tissues in the infected group. The gill tissues were immediately transferred to liquid nitrogen and then stored at −80 °C until extraction and analysis. Three biological replicates were set for each group to reduce errors.

Six cDNA libraries were constructed, including three from the treatment group (T1, T2, T3) and three from the control group (C1, C2, C3), followed by sequencing. Total RNA was extracted from the gill tissues of the infected and control groups using TRIzol reagent (Invitrogen, Carlsbad, CA, USA) according to the manufacturer’s instructions. The quantity and integrity of RNA were evaluated using a microvolume spectrophotometer (Implen, Munich, Germany) and the Agilent 2100 RNA Nano 6000 Assay Kit (Agilent Technologies, Santa Clara, CA, USA). After RNA sample preparation, sequencing libraries were constructed using the NEBNext^®^ Ultra™ RNA Library Prep Kit for Illumina (NEB, Ipswich, MA, USA) according to the manufacturer’s recommendations. First-strand cDNA was synthesized using random primers and M-MuLV Reverse Transcriptase (RNase H^−^; Takara Bio Inc., Shiga, Japan). Second-strand cDNA was synthesized using DNA Polymerase I (Fermentas, Waltham, MA, USA), and the template RNA was hydrolyzed with RNase H. The cDNA fragments in the library were approximately 200–300 bp in length, and the PCR products were purified using the AMPure XP system (Beckman Coulter, Brea, CA, USA). According to the Illumina workflow, index-coded samples were clustered on a cBot Cluster Generation System using the Truseq PE Cluster Kit v3-cBot-HS (Illumina, San Diego, CA, USA) on the Agilent Bioanalyzer 2100 system (Agilent Technologies, Santa Clara, CA, USA). The libraries were sequenced on an Illumina HiSeq platform (Illumina, San Diego, CA, USA) to generate paired-end reads. Quality-controlled reads were aligned to the Poecilia reticulata reference genome (Guppy_female_1.0+MT, NCBI: GCF_000633615.1) using HISAT2 (version 2.2.1) with default parameters. Gene read counts were obtained using HTSeq-count (v0.11.1). The quality assessment of alignment was performed using Qualimap (v2.2.1).

### 2.9. Read Quality Control, Mapping, and Differential Expression Analysis

The raw paired-end reads were trimmed and quality-controlled by Trimmomatic (version 0.36) with parameters (SLIDINGWINDOW:4:15 MINLEN:75). Then, clean reads were aligned to the *Poecilia reticulata* reference genome (Guppy_female_1.0+MT, NCBI: GCF_000633615.1) using HISAT2 (version 2.2.1) software with default parameters. The quality assessment of alignment was performed by Qualimap (version 2.2.1). HTSeq-count (version 0.11.1) was used to count reads mapped to each gene.

To identify differentially expressed genes (DEGs) between the treatment and control groups, edgeR (Empirical Analysis of Digital Gene Expression in R, version 3.40.0) was used for differential expression analysis. The expression level for each gene was calculated using the fragments per kilobase of exon per million mapped reads (FPKM) method for visualization purposes, while raw gene counts were used for statistical analysis. The TMM (Trimmed Mean of M-values) method was applied for normalization. DEGs were selected using the following criteria: |log_2_ fold change| > 1 and false discovery rate (FDR) < 0.05.

### 2.10. Functional Enrichment Analysis

To understand the functions of the differentially expressed genes, GO functional enrichment analysis was carried out using Goatools (version 0.9.9), and KEGG pathway analysis was performed using KOBAS (version 3.0). DEGs were considered significantly enriched in GO terms and metabolic pathways when their Bonferroni-corrected *p*-value was less than 0.05.

### 2.11. Statistical Analysis (Data Are Mean ± SE)

Statistical analyses were performed as follows: in vitro data by one-way ANOVA with Tukey’s test; in vivo survival data by Log-rank (Mantel–Cox) test and Cox proportional hazards regression; the proportional hazards assumption was assessed using Schoenfeld residuals (cox.zph function in R), with no significant violations detected (global test *p* > 0.05) for the 10-day observation period; transcriptomic data by edgeR with |log_2_ fold change| > 1 and FDR < 0.05. Significance was set at *p* < 0.05 or FDR < 0.05.

## 3. Results

### 3.1. Antiparasitic Efficacy of Single Plant Aqueous Extracts

The in vitro test results showed that among the extracts of 10 plant species, 5 exhibited antiparasitic efficacy at a dilution ratio of 1:4 or higher (Table 1). The aqueous extract of *Psoralea corylifolia* was the most effective against *T. pyriformis* among these plants. At a concentration of 100 g/L (1:2 dilution), it only took 1.1 ± 0.1 min to cause 100% death of *T. pyriformis*, and the lethal time prolonged as the concentration was decreased (i.e., the dilution ratio increased). At a concentration of 6.25 g/L (1:32 dilution), the mean duration until death was 88.3 ± 3.1 min. The aqueous extract of *Morus alba* caused a mean death time of 1.8 ± 0.3 min in *T. pyriformis* at a concentration of 100 g/L (1:2 dilution), and when the concentration was decreased to 25 g/L (1:8 dilution), the mean duration until death was 76.2 ± 1.8 min.

The aqueous extract of *Polygonum cuspidatum* resulted in a mean death time of 3.6 ± 0.5 min in *T. pyriformis* at a concentration of 100 g/L (1:2 dilution), and when the concentration was decreased to 25 g/L (1:8 dilution), the mean duration until death was 122.6 ± 2.8 min. The aqueous extracts of *Curcuma longa*, *Sophora flavescens*, and Pomegranate Peel could cause 100% death of *T. pyriformis* within 5 h at a concentration of 50 g/L (1:4 dilution).

The aqueous extracts of *Cynanchum atratum*, *Agrimonia eupatoria*, *Areca*, and *Nelumbo nucifera* still could not cause 100% death of *T. pyriformis* after 5 h of exposure at the concentrations listed in the table, such as 100, 50, 25, 12.5, 6.25, and 3.125 g/L (corresponding to 1:2, 1:4, 1:8, 1:16, 1:32, and 1:64 dilutions, respectively).

### 3.2. Checkerboard Method

The interactions among *Morus alba*, *Polygonum cuspidatum*, *Psoralea corylifolia*, *Sophora flavescens* and Pomegranate Peel were investigated using the checkerboard method (Table 2). The results showed that the combinations of *S. flavescens* + *P. corylifolia* (FIC = 0.5) and *P. corylifolia* + *M. alba* (FIC = 0.5) exhibited a synergistic effect. Additive effects (0.5 < FIC ≤ 1) were observed for *P. cuspidatum* + *P. corylifolia* (FIC = 0.75), *P. cuspidatum* + *M. alba* (FIC = 0.75), *S. flavescens* + *M. alba* (FIC = 1), P. peel + *P. corylifolia* (FIC = 1), and *P. cuspidatum* + *S. flavescens* (FIC = 1). Indifferent effects (1 < FIC ≤ 4) were found in P. peel + *P. cuspidatum* (FIC = 1.5), P. peel + *M. alba* (FIC = 1.25), and P. peel + *S. flavescens* (FIC = 1.25). No antagonistic effects (FIC > 4) were detected in any of the plant combinations (Table 3).

### 3.3. Antiparasitic Effects of Plant Combinations on Tetrahymena pyriformis

Seven combinations with synergistic and additive effects were prepared in equal proportions. In the antiparasitic assay, Combination 6 showed the highest efficacy against the parasites, killing all *T. pyriformis* within 129.0 ± 3.6 min at a concentration of 3.125 g/L (1:64 dilution). It is hypothesized that the flavonoid components in *Psoralea corylifolia* disrupt the cell membrane of the parasite, while the active ingredients in *Morus alba* inhibit its energy metabolism, and their synergistic action accelerates parasite death. Combination 3 and Combination 4 achieved 100% ciliate mortality at a concentration of 6.25 g/L (1:32 dilution) within 150.6 ± 3.2 min and 90.0 ± 2.7 min, respectively (Table 4). During the 5 h exposure period, the minimum lethal concentration (MLC) of Combination 5 against *T. pyriformis* was 12.5 g/L (1:16 dilution), while the MLC of Combination 1 and Combination 2 against ciliates was 25 g/L (1:8 dilution) (Table 4).

### 3.4. Histopathological Characteristics of Poecilia reticulata (Guppy)

**Figure 1:**
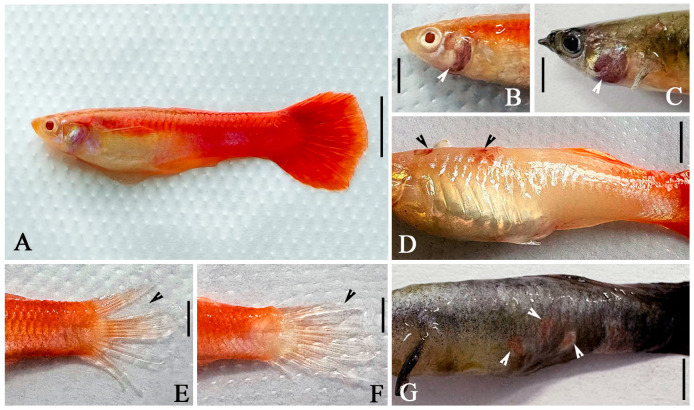
The pathological features of healthy guppy (**A**) and diseased guppy (**B**–**G**). (**A**) Healthy individual of *P. reticulata*. (**E**,**F**) Caudal fin tissue of diseased fish showing detachment (arrowheads). (**B**,**C**) Gills of diseased fish; nearly all diseased fish present with bleeding, redness, and erosion (arrowheads). (**D**,**G**) Diseased fish with red lesions observed across all body parts (arrowheads). Scale bars = 1 cm (**A**), 8 mm (**D**,**G**), and 5 mm (**B**,**C**,**E**,**F**).

**Figure 2:**
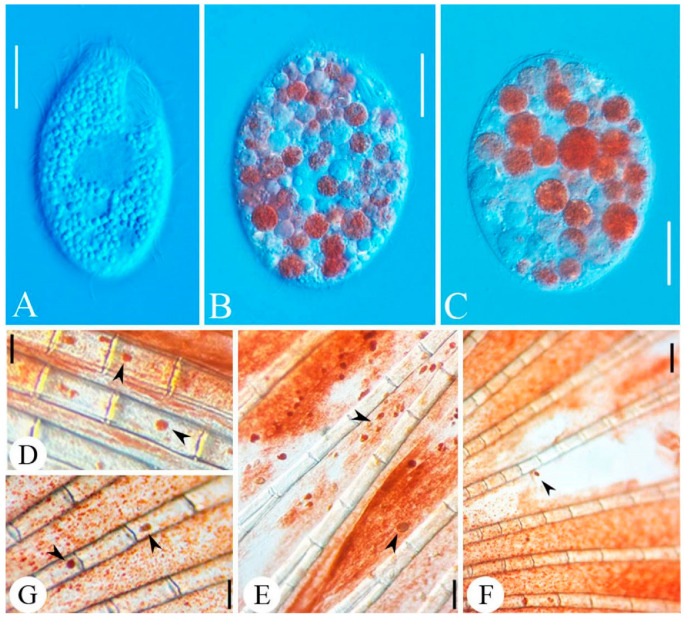
Live photomicrographs of *Tetrahymena pyriformis.* (**A**) Live photomicrograph of *T. pyriformis* that has not yet infected fish. (**B**,**C**) Live photomicrographs of *T. pyriformis* detected on the body surface of diseased fish. (**D**–**G**) Caudal fin of diseased *Poecilia reticulata*; *T. pyriformis* is visible, with ciliates creeping slowly (arrowheads). Scale bars = 10 µm (**A**–**C**) and 80 µm (**D**–**G**).

*Tetrahymena pyriformis* infects the tissues of *P. reticulata* (e.g., gill, liver and muscle tissues), invading through skin lesions and subsequently spreading throughout the body. Infected fish gradually lost vitality, failed to maintain balance, and eventually died. Compared with fish uninfected with *T. pyriformis* (Figure 1A), the gills of almost all diseased fish showed hemorrhage, redness and tissue erosion (Figure 1B,C, arrowheads). In addition, the caudal fins of some guppies were severely damaged with extensive tissue loss (Figure 1E,F, arrowheads). Dark red spots appeared in the subcutaneous tissue of some diseased fish (Figure 1D,G, arrowheads), accompanied by excessive mucus secretion (Figure 1G). Under a light microscope, a large number of *T. pyriformis* were directly observed in fresh samples extracted from gill and fin tissues (Figure 2D–F, arrowheads). Live observation of these *T. pyriformis* cells revealed the presence of fish flesh pigment granules in their food vacuoles (Figure 2B,C).

### 3.5. Evaluation of the Protective Effect of Plant Combinations Against Tetrahymena pyriformis Infection in Poecilia reticulata

**Figure 3:**
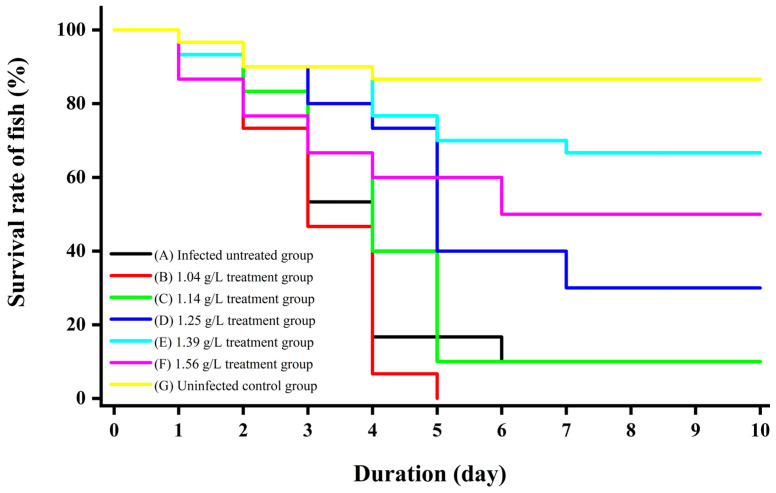
Therapeutic effects of immersion therapy with *P. corylifolia* + *M. alba* on *Tetrahymena pyriformis* infection in guppies (*Poecilia reticulata*) (30 fish per group). (A) Infected untreated group (0 g/L); (B) 1.04 g/L treatment group; (C) 1.14 g/L treatment group; (D) 1.25 g/L treatment group; (E) 1.39 g/L treatment group; (F) 1.56 g/L treatment group; and (G) uninfected control group.

After immersion therapy, the survival rate of *P. reticulata* was observed and recorded. The results showed that the survival rate of the uninfected control group (Group G) remained at a high level throughout the 10-day period, whereas the survival rate of the infected and untreated group (Group A) decreased rapidly after three days. Significant differences were observed in the performance of groups treated with different concentrations of the herbal solution: the 1.39 g/L concentration group (Group E, corresponding to 1:144 dilution) exhibited the relatively optimal protective effect, with a survival rate of 66.7% after 10 days; the survival rate of the 1.56 g/L concentration group (Group F, 1:128 dilution) was finally maintained at approximately 50%; the survival rate of the 1.25 g/L concentration group (Group D, 1:160 dilution) decreased to 30% in the later stage; the survival rates of the 1.14 g/L (Group C, 1:176 dilution) and 1.04 g/L (Group B, 1:192 dilution) concentration groups declined rapidly, with Group C dropping to 0% around the 5th day of the experiment and Group B having a survival rate of 10% at 10 days (Figure 3). Microscopic observation revealed a large number of *T. pyriformis* in the dead fish of Groups B and C, while no *T. pyriformis* were found in the dead fish of Groups F and E.

Statistical analysis using the Log-rank (Mantel–Cox) test demonstrated significant differences in survival distributions among treatment groups (χ^2^ = 119, df = 6, *p* < 0.0001). Cox proportional hazards regression analysis (Table 5) revealed that Groups D (1.25 g/L), E (1.39 g/L), F (1.56 g/L), and G (uninfected control) showed significantly reduced mortality risk compared to the untreated infected control (HR < 1, *p* < 0.001). Notably, Group E (1.39 g/L) exhibited the most pronounced protective effect with a hazard ratio of 0.08 (95% CI: 0.03–0.19, *p* < 0.001). Group F (1.56 g/L) also demonstrated substantial efficacy (HR = 0.14, 95% CI: 0.06–0.32, *p* < 0.001). Conversely, lower concentrations (Groups B and C) showed limited protection, with Group C (1.14 g/L) approaching complete mortality by Day 5 (HR = 0.56, 95% CI: 0.32–0.97, *p* = 0.039) and Group B (1.04 g/L) exhibiting 100% mortality (HR = 0.85, 95% CI: 0.51–1.42, *p* = 0.535).

Based on these survival analyses, when the mortality rate of the infected and untreated group (Group A) approached 50%, fish from the concentration group with the highest survival rate (Group E, 1.39 g/L) were selected as the treatment group (denoted as T1, T2, T3) and untreated diseased fish from Group A as the control group (denoted as C1, C2, C3) for subsequent transcriptomic analysis.

### 3.6. Differentially Expressed Genes (DEGs)

**Figure 4:**
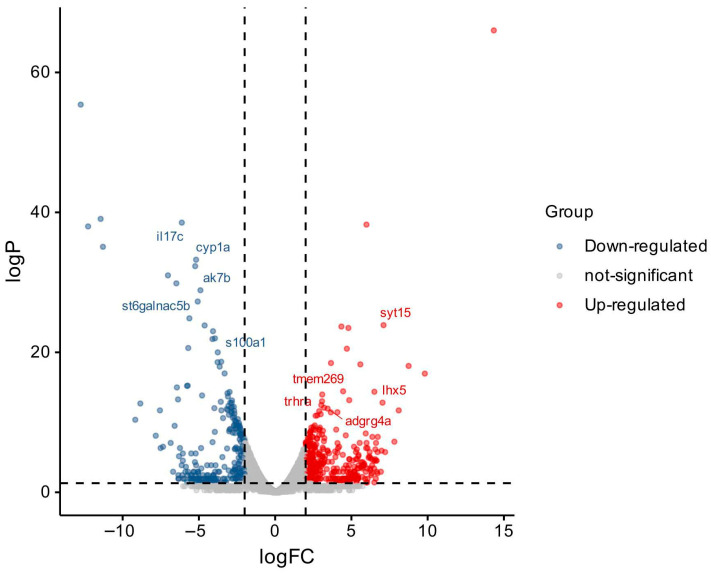
Volcano plot of differentially expressed genes (DEGs) in the gills of diseased fish treated with medicated bath. The horizontal and vertical dotted lines represent the adjusted *p*-value equal to 0.05 and the minimum acceptable fold change, respectively. The log_2_ fold change values of all genes were calculated as log_2_ (treated/control).

Using edgeR (Empirical Analysis of Digital Gene Expression in R, version 3.40.0) with the criteria of |log_2_ fold change| > 1 and false discovery rate (FDR) < 0.05, a total of 577 significantly differentially expressed genes were identified between the treatment group and the control group, among which 228 genes were up-regulated and 349 genes were down-regulated (Figure 4). Genes associated with the immune system and disease processes were identified through Gene Ontology (GO) and Kyoto Encyclopedia of Genes and Genomes (KEGG) analyses. The complete list of all differentially expressed genes, including gene ID, log_2_ fold change, *p*-value, FDR value, expression change type, and gene product description, is provided in Supplementary Table S1.

### 3.7. Gene Ontology (GO) Analysis

**Figure 5:**
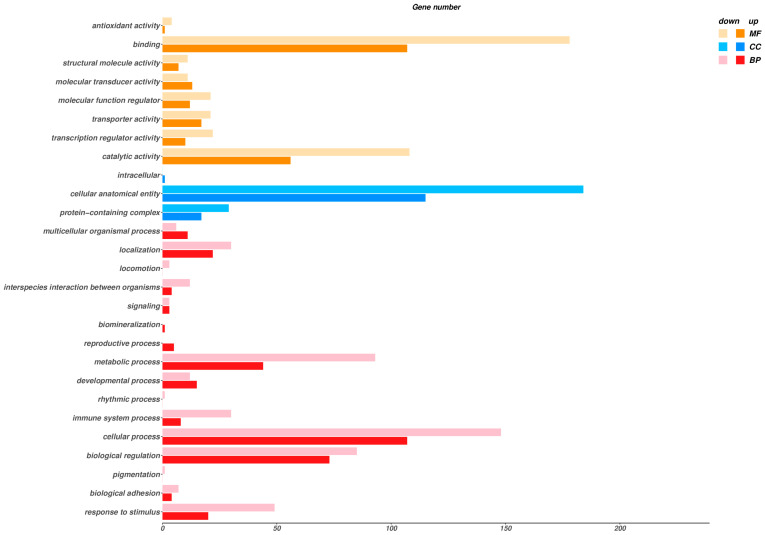
Gene Ontology (GO) annotation of differentially expressed genes (DEGs) in the gills of diseased fish treated with medicated bath.

All differentially expressed genes (DEGs) were classified into three major functional categories based on Gene Ontology (GO) annotations: Biological Process (BP), Cellular Component (CC), and Molecular Function (MF). GO enrichment analysis of all DEGs identified 99 significantly enriched GO terms (68 for Biological Process, 26 for Molecular Function, and 5 for Cellular Component) (adjusted *p*-value ≤ 0.05) (Figure 5). As shown in the figure, DEGs were widely distributed across multiple functional subclasses: in Biological Process, the significantly enriched genes were mainly involved in cellular processes, biological regulation, metabolic processes, response to stimuli, and immune system processes; in Molecular Function, DEGs were primarily associated with binding activity, catalytic activity, transporter activity, and transcriptional regulatory activity; in cellular Component, they were concentrated in cellular anatomical entities, protein complexes, and intracellular structures.

### 3.8. KEGG Pathway Analysis

**Figure 6:**
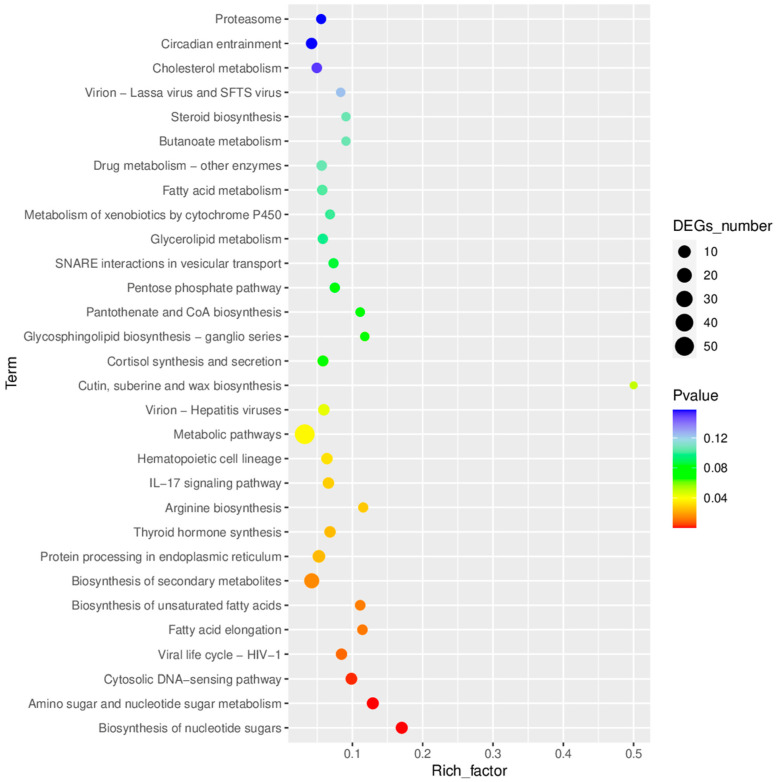
KEGG pathway enrichment analysis of differentially expressed genes (DEGs) in the gills of diseased fish after medicated bath treatment. The top 30 enriched KEGG pathways are displayed, with dot size representing the number of DEGs and color indicating the significance level *(p*-value). Fourteen pathways were significantly enriched (*p* < 0.05), including the IL-17 signaling pathway (6 DEGs), cytosolic DNA-sensing pathway (7 DEGs), amino sugar and nucleotide sugar metabolism (8 DEGs), and metabolic pathways (57 DEGs).

Based on the KEGG database, a Top 30 pathway enrichment analysis was performed on the differentially expressed genes (DEGs) (Figure 6). The enrichment analysis revealed 14 significantly enriched signaling pathways (*p* < 0.05) in the treatment group vs. the control group (T/C group). Among these, 8 (57.1%) were involved in metabolic regulation, immune response, and viral infection-related pathways, indicating that metabolic reprogramming and immune activation are core biological response mechanisms in this comparison group.

Metabolic pathways constituted the largest functional category. Carbohydrate metabolism was significantly represented by amino sugar and nucleotide sugar metabolism (ko00520, 8 DEGs: *gale*, *gmppb*, *gnpnat1*, *nanp*, *nansa*, *pgm3*, *cmah*, *LOC103479017*; *p* = 1.67 × 10^−4^) and biosynthesis of nucleotide sugars (ko01250, 8 DEGs; *p* = 2.16 × 10^−5^). Lipid metabolism pathways included fatty acid elongation (ko00062, 4 DEGs: *acot7*, *elovl1a*, *elovl7a*, *hsd17b12a*; *p* = 0.012) and biosynthesis of unsaturated fatty acids (ko01040, 4 DEGs; *p* = 0.013). Amino acid metabolism was represented by arginine biosynthesis (ko00220, 3 DEGs: *glula*, *lgsn*, *nos1*; *p* = 0.027). Global metabolic pathways such as biosynthesis of secondary metabolites (ko01110, 22 DEGs; *p* = 0.015) and general metabolic pathways (ko01100, 57 DEGs; *p* = 0.038) were also significantly enriched.

Immune-related pathways showed significant enrichment, particularly the IL-17 signaling pathway (ko04657, 6 DEGs: *cebpb*, *fosab*, *fosb*, *mmp13b*, *LOC103470686*, *LOC103471007*; *p* = 0.028) and the cytosolic DNA-sensing pathway (ko04623, 7 DEGs: *LOC103461373*, *LOC103464155*, *LOC103467464*, *LOC103467935*, *LOC103470686*, *LOC103471007*, *LOC103472993*; *p* = 0.002). The hematopoietic cell lineage pathway (ko04640, 6 DEGs: *il11a*, *LOC103456801*, *LOC103470686*, *LOC103473109*, *LOC103478386*, *LOC103480943*; *p* = 0.033) was additionally enriched. Notable transcriptional changes included downregulation of interferon-induced genes (*ifit1*, *ifit2*, *ifit3*, *ifit5*, *ifi44*, *ifi44l*, *mx1*, *mx2*, *mx3*, *isg15*) and pro-inflammatory cytokines (*il17c*, *il1b*), alongside upregulation of genes associated with cellular protection (*mt2*, *hspb6*, *hspb9l*, *sesn1*).

## 4. Discussion

In the aquaculture industry, ciliate parasitic diseases are one of the key bottlenecks restricting the healthy development of the sector [48,49]. Parasitic ciliates represented by *Ichthyophthirius multifiliis* are difficult to effectively control with conventional chemical drugs due to their complex life cycles and persistent infection characteristics. Moreover, chemical treatments pose risks of drug residues, environmental pollution, and the development of drug resistance [20,50,51,52]. Therefore, the development of green, safe, and efficient alternative prevention and control strategies has become an urgent demand in the industry [53]. This study used *T. pyriformis*—a close relative of *I. multifiliis*—as the experimental organism to systematically screen and analyze the antiparasitic effects and mechanisms of several Chinese herbal medicines, providing new insights and experimental evidence for the control of such parasitic diseases. Through in vitro activity screening, combined efficacy verification, and in vivo prevention trials conducted to examine host molecular responses, this study progressively clarified the mode of action of Chinese herbal medicine combinations against ciliates. The results are discussed below in conjunction with existing research.

### 4.1. Analysis of the Activity Screening Results of Single Chinese Herbal Medicines Against Tetrahymena pyriformis

Based on existing research on antiparasitic Chinese herbal medicines, this study screened 10 potentially effective herbs [29,31,54,55,56,57,58,59,60]. Subsequently, in the in vitro antiparasitic screening of single Chinese herbal medicines, we found that the aqueous extracts of five herbs-*Psoralea corylifolia*, *Morus alba*, *Sophora flavescens*, *Polygonum cuspidatum*, and Pomegranate Peel-exerted significant lethal effects on *T. pyriformis*. Among them, *P. corylifolia* and *M. alba* showed the most prominent activity: at a concentration of 100 g/L, they achieved 100% ciliate mortality in only 1.1 ± 0.1 min and 1.8 ± 0.3 min, respectively, with activity significantly higher than the other herbs. Recent studies have found that some plant extracts can kill parasites on fish. For example, extracts from lotus receptacle, soybean seed coat, and agrimony demonstrated good efficacy, with strong killing effects on parasite larvae (EC50 value as low as 2.18 mg/L) [59]. Later research also found that procyanidins from grape seeds demonstrated even greater efficacy, with an EC50 value of only 0.10 mg/L. This substance can damage the parasites’ cilia and mitochondria, causing them to die.

Flavonoids from *P. corylifolia* (such as isobavachalcone) can also directly kill parasites [25]. Flavonoids from mulberry root bark (such as kuwanon G and kuwanon O) also have good antimicrobial effects [31], which is probably why they can kill parasites effectively. In comparison, *Curcuma longa*, *S. flavescens*, and Pomegranate Peel were less effective, needing a higher concentration (1:4) and 5 h of exposure to kill all the parasites. The other five herbs (such as *Cynanchum atratum* and *Agrimonia eupatoria*) could not kill the parasites even at a high concentration (1:2). This shows that different Chinese herbs have very different effects on killing parasites, which may be related to the types and amounts of active ingredients in the herbs, as well as parasite sensitivity to the compounds [61].

### 4.2. Synergistic/Additive Effects and Mechanisms of Herbal Combinations

Using the checkerboard assay, we systematically analyzed pairwise combinations of the five highly active Chinese herbal medicines. The results showed that multiple compatibility schemes could effectively enhance antiparasitic efficacy. In particular, two combinations—*P. corylifolia* + *M. alba* and *P. corylifolia* + *S. flavescens*—exhibited synergistic effects (FIC = 0.5). Additionally, four combinations showed additive effects (0.5 < FIC ≤ 1), including *P. cuspidatum* + *P. corylifolia*, *P. cuspidatum* + *M. alba*, *S. flavescens* + *M. alba*, and *P. cuspidatum* + *S. flavescens*. Among these, the combination of *P. corylifolia* and *M. alba* showed the most remarkable synergy with an FIC index of 0.5. Additionally, three combinations displayed additive effects, and no antagonistic effects were observed in any of the tested combinations. The synergistic effect may arise from two mechanisms: first, different active ingredients act on multiple targets of the parasite, respectively. For example, pentagalloyl glucose mainly causes parasite swelling and death by disrupting the cell membrane, increasing intracellular osmotic pressure, and promoting water influx into the cell [62]; quinine can alter the food vacuoles and contractile vacuoles of *I. multifiliis*, impairing its digestive and osmoregulatory functions and ultimately leading to parasite death [63]. Therefore, it is speculated that the synergistic effect may arise from two mechanisms. First, psoralen from *P. corylifolia* and flavonoids from *M. alba* root bark may disrupt the parasite’s cell membrane structure and interfere with its energy metabolism, thereby accelerating parasitic death through multi-target action. Second, certain components in the combination may promote the dissolution, absorption, or transformation of other active ingredients, thereby increasing the drug concentration at the site of action. It is important to note that the transition from volume-optimized in vitro combinations to mass-based in vivo preparations assumes comparable extraction efficiencies between the two botanical species; future studies should verify bioequivalence through analytical quantification of marker compounds in the final decoctions. Previous studies also support this finding. It has been reported that the combination of *Cynanchum atratum* and *P. corylifolia* showed significantly better lethal effects on *I. multifiliis* than either single drug [56], and this synergy was also attributed to the interaction between components. This study further confirms that rational compatibility of Chinese herbal medicines can indeed achieve a “1 + 1 > 2” synergistic effect in ciliate control, expanding the application prospects of Chinese herbal medicines in aquatic parasite prevention and control.

### 4.3. Protective Effect of Psoralea corylifolia–Morus alba Combination on Guppies In Vivo

In the in vivo experiments using infected models, the combination of *P. corylifolia* and *M. alba* exhibited obvious preventive and therapeutic effects in a concentration-dependent manner. When guppies infected with *T. pyriformis* were treated with a 1:144 dilution bath, the highest survival rate (66.7%) was achieved within 10 days, and no viable ciliates were detected in the dead fish. This result indicates that the in vivo efficacy is closely related to the concentration of Chinese herbal medicines, and either excessively high or excessively low concentrations will affect the preventive and therapeutic efficacy [64]. Histopathological observations further confirmed the above conclusion. Obvious damage, including hemorrhage and erosion, was observed in the gill tissues of infected fish, accompanied by a large number of attached ciliates. In contrast, the pathological damage to the gill tissues of fish treated with the effective concentration was significantly alleviated. This suggests that the combination not only effectively kills ciliates but also exerts a protective effect on host tissues. Furthermore, fish flesh pigment granules were observed in the food vacuoles of infected parasites. This finding directly confirms morphologically that *T. pyriformis* can cause physical damage to guppies through phagocytosis, providing intuitive evidence for understanding its pathogenic mechanism.

### 4.4. Host Immune and Metabolic Regulatory Mechanisms Revealed by Transcriptomic Analysis

Through gill tissue transcriptome analysis, we revealed the core pathways by which the Chinese herbal medicine combination regulates the host’s antiparasitic response at the molecular level. A total of 577 differentially expressed genes (DEGs) were identified between the treatment group and the control group, of which 228 were upregulated and 349 were downregulated. Functional enrichment analysis showed that these genes are mainly involved in two aspects: metabolic regulation and immune response. In terms of immunity, the IL-17 signaling pathway and cytosolic DNA-sensing pathway were significantly activated. The IL-17 pathway is an important pathway for vertebrates to resist parasitic infections; its activation can promote the release of inflammatory factors such as IL-6 and TNF-α [65,66,67,68], enhancing the mucosal immune barrier function. The activation of the cytosolic DNA-sensing pathway suggests that after the host recognizes ciliate DNA, it may indirectly exert antiparasitic effects by inducing antiviral factors such as IFN-γ [69,70,71]. This finding suggests that the combination of *P. corylifolia* and *M. alba* may enhance host anti-infection capacity through modulation of innate immune pathways, though whether these effects represent direct pharmacological immunostimulation or secondary recovery responses following parasite elimination requires further validation. Notably, the observed immune activation, if pharmacologically driven, could theoretically strengthen mucosal barrier integrity and reduce parasite adhesion—mechanisms requiring validation in controlled follow-up studies.

The alterations in metabolism-related pathways reveal a novel mechanism by which herbal medicine combinations assist the host in adapting to infection and maintaining metabolic homeostasis. The study found that pathways such as amino sugar metabolism and fatty acid elongation were significantly regulated: the activation of the amino sugar metabolism pathway can promote the synthesis of glycoproteins in the gill mucosa. As key components of the physical barrier in the host mucosa, the increased synthesis of glycoproteins can enhance the defensive capacity of gill tissues against parasitic invasion and reduce parasite adhesion and penetration [72,73]. The upregulation of the fatty acid synthesis pathway can provide the necessary energy supply and material substrates for immune-related processes such as immune cell proliferation, differentiation, and cytokine secretion, thereby laying a metabolic foundation for the efficient initiation and sustained exertion of the host’s anti-parasitic immune response [74]. This finding indicates that the *P. corylifolia*–*M. alba* combination may not only exert direct parasiticidal effects but also potentially modulate the host’s core metabolic pathways to construct a “metabolism-immunity” coordinated response network, though it remains unclear whether these metabolic changes represent direct pharmacological intervention or natural physiological recovery following parasite clearance. The enrichment of amino sugar metabolism genes (*gale*, *gmppb*, *pgm3*) specifically suggests potential for mucin glycoprotein upregulation, which—if confirmed at the protein level—could create steric hindrance against ciliate attachment, representing a host-directed defense mechanism complementary to direct parasiticidal action.

### 4.5. Study Limitations

Several critical limitations of this study must be acknowledged. First, the reliance on crude aqueous extracts without rigorous phytochemical fingerprinting (e.g., HPLC or LC-MS analysis) means the specific molar concentrations of bioactive metabolites—such as psoralen, isobavachalcone, and kuwanon G—remain unknown. This chemical ambiguity limits reproducibility and industrial standardization. Second, while CHMs are promoted as eco-friendly alternatives, the potential ecological toxicity of the 1:144 dilution to non-target aquatic organisms, particularly nitrifying bacteria in biofiltration systems, remains uncharacterized. Third, the transcriptomic findings rely solely on RNA-seq without orthogonal validation via quantitative real-time PCR, introducing bioinformatic uncertainty regarding precise fold-change magnitudes. Fourth, *T. pyriformis* lacks the complex, multi-stage life cycle of *I. multifiliis*, particularly the resilient encysted tomont stage that is impermeable to most therapeutics. Consequently, the efficacy observed against this single-stage, free-swimming model may not translate directly to clinical white spot disease outbreaks, as the herbal extract may fail to penetrate the protective cyst wall of *I. multifiliis*.

## 5. Conclusions

**Figure 7:**
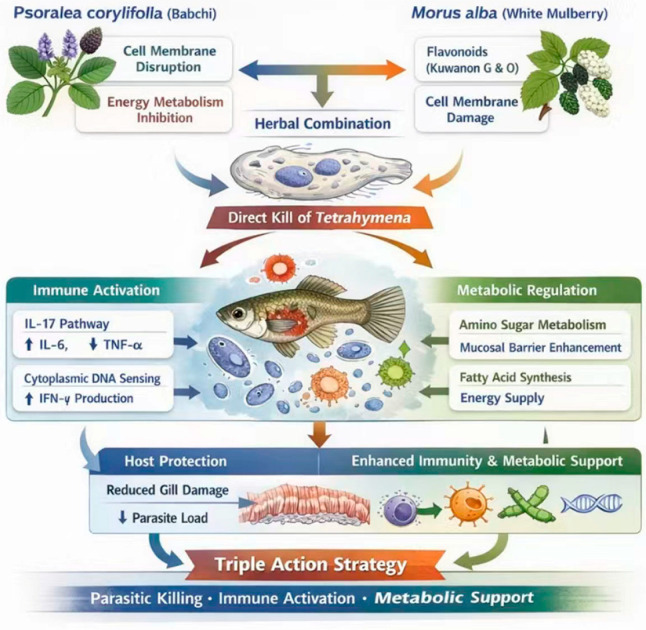
Schematic diagram illustrating the mechanism and protective effects of *Psoralea corylifolia-Morus alba* combination against *Tetrahymena pyriformis* infection.

This study demonstrates that the combination of *P. corylifolia* and *M. alba* exerts potent antiparasitic effects against *T. pyriformis* in vitro and improves survival in infected guppies. The treatment is associated with alterations in host gene expression related to immune pathways (IL-17 and cytosolic DNA-sensing) and metabolic processes (amino sugar and fatty acid metabolism), though whether these represent direct pharmacological effects or secondary recovery responses requires further investigation (Figure 7). These findings suggest there is potential for developing plant-based alternatives to chemical drugs for ciliate disease control.

The *P. corylifolia*–*M. alba* combination, particularly at a 1:144 dilution, may be considered as a candidate bath treatment for *T. pyriformis* infections in guppies. Its direct antiparasitic activity is well demonstrated, while its effects on host immune and metabolic pathways warrant additional controlled studies to establish causality. Further research is needed to evaluate comparative efficacy, long-term safety, and environmental impact relative to chemical treatments.

Future studies should address (i) phytochemical standardization to identify active compounds; (ii) mechanistic validation of immunomodulatory effects through appropriate control groups; (iii) toxicity assessment in target and non-target organisms; and (iv) efficacy testing against *I. multifiliis*, noting that the current model (*T. pyriformis*) lacks the resilient encysted stage characteristic of white spot disease.

## Figures and Tables

**Table 1:** Lethal time of aqueous extracts from ten plant species on *Tetrahymena pyriformis*.

Extract	Mean Duration Until Death of All *T. pyriformis* (min)						
	100 g/L	50 g/L	25 g/L	12.5 g/L	6.25 g/L	3.125 g/L	0
*Curcuma longa*	103.0 ± 2.6	298.5 ± 4.4	-	-	-	-	-
*Cynanchum atratum*	-	-	-	-	-	-	-
*Morus alba*	1.8 ± 0.3	33.6 ± 1.3	76.2 ± 1.8	-	-	-	-
*Psoralea corylifolia*	1.1 ± 0.1	5.2 ± 0.6	13.4 ± 0.9	44.0 ± 1.4	88.3 ± 3.1	-	-
*Sophora flavescens*	14.4 ± 2.1	58.6 ± 3.5	-	-	-	-	-
*Agrimonia eupatoria*	-	-	-	-	-	-	-
*Areca*	-	-	-	-	-	-	-
*Polygonum cuspidatum*	3.6 ± 0.5	58.9 ± 2.1	122.6 ± 2.8	-	-	-	-
Pomegranate Peel	8.7 ± 1.0	279.4 ± 2.6	-	-	-	-	-
*Nelumbo nucifera*	-	-	-	-	-	-	-

-: The extract at the listed concentration could not kill 100% *T. pyriformis* by 5 h exposure. Concentrations are given as g/L of herbal extract in the test solution, corresponding to dilutions of the stock solution (200 g/L). Values are expressed as means ± standard error of the mean (SEM) of three replicates.

**Table 2:** Five plants with high antiparasitic efficacy against *Tetrahymena pyriformis* were selected and combined at a ratio of 1:1 (m:m).

		Plant A (Columns)				
		*Morus alba*	*Psoralea corylifolia*	*Sophora flavescens*	*Polygonum cuspidatum*	Pomegranate Peel
Plant B (Rows)	*M. alba*	-	-	-	-	-
	*P. corylifolia*	PCO+MA	-	-	-	-
	*S. flavescens*	SF+MA	SF+PCO	-	-	-
	*P. cuspidatum*	PCU+MA	PCU+PCO	PCU+SF	-	-
	P. Peel	PP+MA	PP+PCO	PP+SF	PP+PCU	-

MA: *Morus alba*; PCO: *Psoralea corylifolia*; SF: *Sophora flavescens*; PCU: *Polygonum cuspidatum*; PP: Pomegranate Peel. Plant A refers to the medicinal plant listed in the column headers, and Plant B refers to the medicinal plant listed in the row headers. Each cell represents the combination of the corresponding Plant A and Plant B at a 1:1 mass ratio. “-“ indicates that the combination was not tested or is the same plant self-combination.

**Table 3:** Assessment of plant combinations with checkerboard array method.

Plant Combination	MIC A in Combination (g/L)	MIC A Alone (g/L)	MIC B in Combination (g/L)	MIC B Alone (g/L)	FIC Index	Interaction
PP+PCU	25	50	12.5	12.5	1.5	Indifference
SF+MA	12.5	25	6.25	12.5	1	Additive
SF+PCO	6.25	25	1.56	6.25	0.5	Synergistic
PP+MA	12.5	50	12.5	12.5	1.25	Indifference
PCU+PCO	6.25	12.5	1.56	6.25	0.75	Additive
PP+PCO	25	50	3.125	6.25	1	Additive
PCU+MA	6.25	12.5	3.125	12.5	0.75	Additive
PCO+MA	1.56	6.25	3.125	12.5	0.5	Synergistic
PP+SF	12.5	50	25	25	1.25	Indifference
PCU+SF	6.25	12.5	12.5	25	1	Additive

MA: *Morus alba*; PCO: *Psoralea corylifolia*; SF: *Sophora flavescens*; PCU: *Polygonum cuspidatum*; PP: Pomegranate Peel. FIC: Fractional inhibitory concentration index; FIC index = MIC A in combination/MIC A alone + MIC B in combination/MIC B alone; Synergism, FIC ≤ 0.5; Additive effect, 0.5 < FIC ≤ 1; Indifference, 1 < FIC ≤ 4; Antagonism, FIC > 4.

**Table 4:** Antiparasitic effect of plant combinations on the survival of *Tetrahymena pyriformis*.

Extract	Mean Duration Until Death of All *T. pyriformis* (min)						
	50 g/L	25 g/L	12.5 g/L	6.25 g/L	3.125 g/L	1.56 g/L	0
Com 1	6.3 ± 0.6	24.1 ± 1.4	-	-	-	-	-
Com 2	11.2 ± 1.0	120.0 ± 2.7	-	-	-	-	-
Com 3	8.0 ± 0.9	15.2 ± 1.0	50.3 ± 1.7	150.6 ± 3.2	-	-	-
Com 4	5.7 ± 0.6	16.6 ± 1.3	31.2 ± 2.3	90.0 ± 2.7	-	-	-
Com 5	10.5 ± 1.2	33.7 ± 1.3	126.9 ± 2.0	-	-	-	-
Com 6	4.1 ± 0.3	9.8 ± 0.6	16.5 ± 1.0	54.3 ± 1.3	129.0 ± 3.6	-	-
Com 7	15.0 ± 1.2	68.6 ± 1.7	-	-	-	-	-

Com 1: Sophora flavescens and Morus alba; Com 2: S. flavescens and Psoralea corylifolia; Com 3: Polygonum cuspidatum and P. corylifolia; Com 4: Pomegranate Peel and P. corylifolia; Com 5: P. cuspidatum and M. alba; Com 6: P. corylifolia and M. alba; Com 7: P. cuspidatum and S. flavescens; -: Plant combination at the listed concentrations did not kill all T. pyriformis after 5 h exposure. Values are expressed as means ± standard error of the mean (SEM) of three replicates.

**Table 5:** Hazard ratios for mortality of guppies treated with different dilutions of *P. corylifolia* + *M. alba* combination.

Group	Concentration (g/L)	HR	95% CI	*p*-Value
A	Untreated	1.00	–	–
B	1.04	0.85	0.51–1.42	0.535
C	1.14	0.56	0.32–0.97	0.039
D	1.25	0.23	0.11–0.46	<0.001
E	1.39	0.08	0.03–0.19	<0.001
F	1.56	0.14	0.06–0.32	<0.001
G	Uninfected	0.06	0.02–0.17	<0.001

HR < 1 indicates reduced mortality risk compared to untreated control (Group A). CI: confidence interval. Overall log-rank test: χ^2^ = 119, df = 6, and *p* < 0.0001.

## Data Availability

Additional data are available upon request from the corresponding authors.

## Supplementary Materials

The following supporting information can be downloaded at: https://www.mdpi.com/article/10.3390/ani16060979/s1, Table S1: T_vs_C.fpkm.DEG.
